# Dual-SwinOrd: A Dual-Head Swin Transformer with Semantic Prior Injection for Ordinal Diabetic Retinopathy Grading

**DOI:** 10.3390/bioengineering13040374

**Published:** 2026-03-24

**Authors:** Wenjuan Yu, Xiaonan Si, Jingxiang Zhong

**Affiliations:** 1 The First Affiliated Hospital of Jinan University, Guangzhou 510630, China; wenjuanyu@stu2023.jnu.edu.cn; 2 The School of Biomedical Engineering, University of Science and Technology of China, Hefei 230027, China; sxnyyds@mail.ustc.edu.cn; 3 Department of Ophthalmology, Shenzhen Bright Star Eye Hospital, Shenzhen 518002, China

**Keywords:** Diabetic Retinopathy, Swin Transformer, Dual-Head Ordinal Regression, Vision–Language Priors, PubMedCLIP, Lesion-aware Attention

## Abstract

Diabetic retinopathy (DR) is the largest cause of permanent vision loss in the working-age population, making automated grading critical for timely therapeutic intervention. While recent deep learning algorithms have improved feature discrimination, modern state-of-the-art systems have two fundamental drawbacks. First, most models rely on standard Convolutional Neural Networks, which struggle to capture long-range relationships and lack semantic reasoning, resulting in visual findings that do not correlate with clinical knowledge. Second, present approaches often consider grading as a nominal classification or a pure ordinal regression task, failing to strike a compromise between high classification accuracy and severity-consistent predictions (Quadratic Weighted Kappa). To address these challenges, we propose Dual-SwinOrd, a novel framework that integrates a hierarchical Vision Transformer with a semantically guided dual-head mechanism. Specifically, we use a Swin Transformer backbone to extract hierarchical features, effectively capturing global retinal structures. To handle diverse lesion scales, we incorporate a Progressive Lesion-aware Kernel Attention (PLKA) module and a Semantic Prior Modulation (SPM) module guided by PubMedCLIP, bridging the gap between visual features and medical linguistic priors. In addition, we propose a Dual-Head learning strategy that decouples the optimization objective into two parallel streams: a Classification Head to maximize diagnostic accuracy and an Ordinal Regression Head (DPE) to enforce rank-consistency. This design effectively mitigates the trade-off between precision and ordinality. Extensive experiments on the APTOS 2019 and DDR datasets demonstrate that Dual-SwinOrd achieves state-of-the-art performance, yielding an Accuracy of 87.98% and a Quadratic Weighted Kappa (QWK) of 0.9370 on the APTOS 2019 dataset, as well as an Accuracy of 86.54% and a QWK of 0.9040 on the DDR dataset.

## 1. Introduction

Diabetic Retinopathy (DR) is a chronic, progressive microvascular consequence of diabetes mellitus that is still the largest cause of permanent blindness in the working-age population worldwide. Diabetes prevalence is expected to rise by 2045, leading to a considerable increase in DR patients [1,2]. DR development is clinically classified into five ordinal severity levels: no DR, mild, moderate, severe, and proliferative DR (PDR), characterized by particular lesions such as microaneurysms, hemorrhages, and neovascularization [3]. Early diagnosis and exact grading are crucial, since timely treatments can minimize the likelihood of severe vision loss by almost 90%. However, because retinal diseases are complicated and multi-scale, manual grading is primarily dependent on ophthalmologists’ subjective knowledge and is prone to substantial inter-observer variability. As a result, developing automated, accurate, and resilient computer-aided diagnosis (CAD) systems has become an essential research goal [4].

Deep learning has transformed automated DR screening. Early frameworks used Convolutional Neural Networks (CNNs), including ResNet [5] and DenseNet [6], to extract visual representations. Also, recent studies have explored promising bio-inspired machine learning approaches for type 2 diabetes and related complication detection [7]. To improve feature discriminability, attention techniques such as CBAM [8] and non-local blocks [9] were developed to capture long-range dependencies. Despite their success, CNN-based approaches have a basic limitation: convolution operations’ fixed receptive field limits their capacity to simulate global retinal structures and long-range semantic connections adequately. Vision Transformers (ViTs), specifically the Swin Transformer, have recently emerged as a powerful alternative, allowing hierarchical feature extraction with shifted window attention that captures both local details and global context, superior to typical CNNs.

However, using Transformers to DR grading still presents two significant issues that compromise clinical dependability. First, there is a clear “semantic gap”. Most advanced models rely only on data-driven visual pattern matching [10], without high-level semantic reasoning to correlate visual discoveries with medical knowledge. They treat lesions as pixel clusters rather than pathogenic signals as specified in the clinical literature. Second, there is a conflict between Classification Accuracy and Ordinal Consistency. DR grading is essentially an ordinal regression task (0 < 1 < 2 < 3 < 4). Standard Cross-Entropy loss improves nominal accuracy but ignores severity, resulting in clinically harmful errors (for example, misclassifying PDR as Healthy). Pure ordinal regression approaches frequently prioritize rank consistency over fine-grained classification accuracy [11]. Existing single-head designs fail to balance these two opposing goals simultaneously.

To address these challenges, we propose Dual-SwinOrd, a novel framework that synergizes a Swin Transformer backbone with a dual-head learning strategy. Unlike prior techniques, which just deepened CNNs, we introduce the Swin Transformer to capture hierarchical global characteristics. To fill the semantic vacuum, we propose a Semantic Prior Modulation (SPM) module driven by PubMedCLIP [12] that injects expert-level clinical semantics into visual representations. To deal with the significant scale diversity of lesions (from microscopic microaneurysms to huge hemorrhages), we propose a Progressive Lesion-aware Kernel Attention (PLKA) mechanism. Most importantly, to address the accuracy-ordinality trade-off, we propose a Dual-Head design. This design separates the optimization into two simultaneous streams: a Classification Head that maximizes diagnostic precision and an Ordinal Head that reduces severity estimation risk using a Cost-sensitive Adaptive Risk Minimization (CARM) loss. The main contributions of this paper are summarized as follows:

- We first integrate biomedical semantic priors into Diabetic Retinopathy grading. To the best of our knowledge, this is one of the first works to utilize a pre-trained Vision–Language Model to guide a Swin Transformer. This design effectively helps reduce the gap between visual representations and clinically meaningful concepts, providing additional semantic guidance beyond purely visual pattern recognition.
- We propose Dual-SwinOrd, a unified framework featuring a novel Dual-Head Strategy. By decoupling the learning objectives into a Classification Head and an Ordinal Head, we successfully mitigate the long-standing trade-off between diagnostic Accuracy and ordinal consistency (Kappa), achieving state-of-the-art performance in both metrics simultaneously.
- We design the Progressive Lesion-aware Kernel Attention (PLKA) module to handle multi-scale challenges. Unlike fixed-size convolutions, PLKA employs dynamic multi-branch kernels to adaptively capture both microscopic lesions and large-scale anomalies, significantly enhancing feature representation.
- Extensive experiments on the DDR and APTOS 2019 datasets demonstrate the superiority of our approach. Dual-SwinOrd outperforms existing CNN-based and ensemble baselines, achieving an accuracy of 87.98% and a QWK of 0.9370 on the APTOS 2019 benchmark. This sets a new benchmark for interpretable, robust, and clinically reliable DR screening.

The remainder of this paper is organized as follows: Section 2 reviews related work. Section 3 details the proposed methodology. Section 4 presents the experimental results, followed by a discussion in Section 5 and conclusions in Section 6.

## 2. Related Work

The application of deep learning and machine learning has shown immense potential across various medical domains. For instance, chronic kidney disease and Gastrointestinal Lesion detection [13,14]. For the past decade, Convolutional Neural Networks (CNNs) have been the foundation of automated DR screening. Early efforts used common architectures like ResNet and DenseNet to extract visual representations [5,6]. Researchers experimented with different attention techniques to improve feature discriminability. For example, Woo et al. [8] proposed CBAM to calibrate channel and spatial features, and Madarapu et al. [9] combined non-local blocks to capture long-range relationships. Khan et al. [10] and Bodapati et al. [15] utilized evolutionary algorithms and adaptive weighting to combine predictions from multiple CNN backbones, improving robustness against inter-patient variability.

Despite their advances, CNN-based approaches have narrow receptive fields, making it challenging to describe global retinal structures and long-term semantic relationships. Recently, Vision Transformers (ViTs) have emerged as a viable option. Unlike CNNs, ViTs use self-attention mechanisms to understand global context. The Swin Transformer [16] utilized hierarchical feature maps and shifted window focus to effectively balance local feature extraction with global modeling. While Swin Transformers have demonstrated better performance in general medical imaging, their use to fine-grained, multi-scale DR grading is yet underexplored, especially when combined with domain-specific components.

DR grading is fundamentally an ordinal regression task (0 < 1 < 2 < 3 < 4). However, most existing frameworks treat it as a nominal multi-class classification problem optimized via Cross-Entropy loss [17]. While CE loss is effective for maximizing accuracy, it ignores the severity distance between classes, potentially treating a critical misclassification the same as a minor error. To address this, several works have adopted pure ordinal regression approaches [11,18], which learn a series of binary sub-tasks to enforce rank consistency. However, a critical trade-off exists: pure ordinal models often achieve high Quadratic Weighted Kappa but may sacrifice fine-grained Classification Accuracy compared to CE-based models. Recent trends in multi-task learning suggest that decoupling these objectives can offer the best of both worlds. Our work builds upon this insight by proposing a Dual-Head strategy, which jointly optimizes a classification head for precision and an ordinal head for severity consistency, effectively resolving the trade-off found in single-task networks.

Traditional computer vision models have a “semantic gap,” meaning they rely entirely on pixel patterns without comprehending clinical pathology. Recent studies have shown that Vision–Language Models (VLMs) such as CLIP [19] can effectively align visual features with verbal semantics. In the medical domain, modified models such as MedCLIP [20] and PMC-CLIP [21] use large-scale biomedical image-text pairs to learn robust representations. PubMedCLIP [12] is particularly relevant, as it is fine-tuned on the ROCO dataset and biomedical literature, capturing specialized medical terminology better than generic models. Despite these developments, most current DR grading systems are uni-modal (vision-only). They pass up the potential to use rich clinical priors—such as textual descriptions of “microaneurysms” or “venous beading”—to guide the visual framework. Our method overcomes this issue by explicitly integrating PubMedCLIP-derived semantic priors into the visual pipeline using a gating mechanism.

Despite significant progress, current DR grading systems have three fundamental limitations: (1) CNN-based backbones struggle to capture long-range dependencies necessary for modeling global retinal structures; (2) vision-only models lack the high-level semantic reasoning required to interpret pathological findings; and (3) single-head architectures frequently fail to balance precise classification accuracy with ordinal consistency. To address these gaps, we offer Dual-SwinOrd, a unified framework that combines a hierarchical Swin Transformer with a unique Dual-Head Strategy. Our technique improves feature representation globally and locally by merging PubMedCLIP-guided semantic priors via the SPM module with multi-scale lesion awareness via the PLKA module. Furthermore, the dual-head mechanism decouples the learning objectives, enabling simultaneous optimization of diagnostic precision and severity-aware ranking, setting a new standard for robust and interpretable DR grading.

## 3. Materials and Methods

This section describes the architecture and components of the proposed Dual-SwinOrd framework. Section 3.1 describes the datasets and the preprocessing steps. Section 3.2 presents an overview of the network. The core components are detailed in the following sections: the hierarchical Swin Transformer backbone (Section 3.3), the Biomedical Semantic Prior Modulation (SPM) module (Section 3.4), the Progressive Lesion-aware Kernel Attention (PLKA) module (Section 3.5), and the Dual-Head Learning Strategy formulation (Section 3.6).

### 3.1. Datasets and Preprocessing

To evaluate the clinical robustness and generalization capability of the proposed method, we used two widely renowned public benchmarks: the APTOS 2019 Blindness Detection dataset and the Diabetic Retinopathy Dataset (DDR). Both datasets follow the worldwide clinical grading standard, which divides DR severity into five ordinal scales: No DR (0), Mild (1), Moderate (2), Severe (3), and Proliferative (4).

APTOS 2019 [22] consists of 3662 retinal fundus photos obtained from India’s Aravind Eye Hospital. These photos were taken using several fundus photography devices under a variety of imaging settings, which presented obstacles such as illumination differences and artifacts. For our experiments, we used a randomized split of 80% training and 20% testing.

DDR [23] is a large-scale, demanding dataset of 13,673 pictures collected from 147 hospitals in 23 Chinese regions. Unlike APTOS, DDR has a predefined official division and includes photos of varying resolutions and viewing angles, making it an excellent benchmark for assessing fine-grained lesion identification performance.

As illustrated in Table 1, both datasets exhibit significant class imbalance, with the majority of samples falling into the “No DR” category. This distribution disparity poses a critical challenge for standard classifiers and necessitates the use of our proposed Cost-sensitive Adaptive Risk Minimization (CARM) loss to prevent bias toward majority classes.

### 3.2. Overview of Dual-SwinOrd

**Figure 1:**
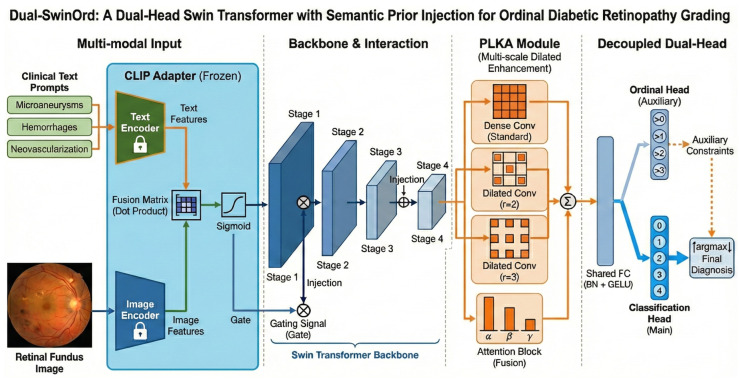
Overall architecture of the proposed Dual-SwinOrd framework for ordinal diabetic retinopathy grading. The network comprises four key components: (1) Multi-modal Input& Interaction: A frozen text adapter extracts semantic priors from clinical text prompts (e.g.,“Microaneurysms”) and retinal fundus images. These priors are integrated via a fusion matrix that produces a gating signal through sigmoid activation. This signal is injected into the Swin Transformer backbone to guide visual feature extraction. (2) Backbone: A hierarchical Swin Transformer extracts multi-scale visual features across four stages (Stage 1 to Stage 4). (3) PLKA Module: The Parallel Large-Kernel Attention (PLKA) module enhances contextual representation using parallel convolutional branches with different dilation rates (standard, r=2, r=3), followed by an attention-based fusion mechanism. (4) Decoupled Dual-Head: A shared fully connected layer feeds into two branches: an auxiliary Ordinal Head that enforces rank-consistent constraints (i.e., predicting whether severity > *k*), and a main Classification Head that outputs the final diagnosis probabilities. The final prediction is derived via argmax over the classification output, refined by the ordinal constraints.

We propose Dual-SwinOrd, a deep integrative framework designed to bridge the semantic gap and resolve the accuracy-ordinality trade-off in Diabetic Retinopathy (DR) grading. As illustrated in Figure 1, the framework comprises four synergistic components: (1) A Swin Transformer Backbone extracts hierarchical visual representations with global context. (2) A PubMedCLIP-powered Biomedical Semantic Prior Modulation (SPM) module injects domain-specific clinical knowledge. (3) A Progressive Lesion-aware Kernel Attention (PLKA) module captures multi-scale pathological features. (4) A novel Dual-Head Learning Strategy optimizes diagnostic accuracy and ordinal consistency.

### 3.3. Hierarchical Swin Transformer Backbone

We use the Swin Transformer as our visual encoder, which differs from standard CNN-based models that use fixed receptive fields. DR lesions, such as microaneurysms and hemorrhages, are spread across the retina, necessitating a model that can capture both local fine-grained details and global geometric patterns. The Swin Transformer creates a hierarchical feature map by merging picture patches in deeper levels. It employs Shifted Window Multi-head Self-Attention (SW-MSA) to enable cross-window connections while retaining linear computing complexity. Given an input fundus image $I\in R^{H\times W\times 3}$, the backbone outputs a feature map $F_{vis}\in R^{C\times \frac{H}{32}\times \frac{W}{32}}$, where *C* is the channel dimensions. This hierarchical architecture serves as a solid platform for future semantic and multi-scale upgrades. When training very deep multi-layer neural networks, models frequently experience the vanishing gradient problem, which prevents effective weight updates and degrades feature learning. To address this and ensure stable convergence throughout the deep hierarchical structure, advanced optimization techniques, such as the oriented stochastic loss descent algorithm, can be used to efficiently train deep networks without vanishing gradients.

### 3.4. Biomedical Semantic Prior Modulation (SPM)

Standard visual backbones frequently fail to account for high-level clinical semantics. We propose the SPM module to overcome this “semantic gap” by using linguistic priors from biomedical literature. We use PubMedCLIP [12], a pre-trained vision–language model for biomedical texts, to extract semantic embeddings. Let $T=\{T_{0},T_{1},\ldots ,T_{K-1}\}$ be a set of expert-curated prompts matching to diagnostic markers (e.g., “Fundus image with microaneurysms” and “Proliferative neovascularization”). The PubMedCLIP image encoder produces a global visual embedding $v\in R^{D}$, whilst the text encoder produces text embeddings $T_{txt}\in R^{K\times D}$ (D=512). We calculate a similarity-based attention distribution.

(1) $$W_{sem}=\mathrm{Softmax}\left(\frac{v\cdot T_{txt}^{⊤}}{\tau }\right)\in R^{K}$$

where τ is a temperature parameter. The aggregated semantic context vector is:

(2) $$C_{sem}=W_{sem}\cdot T_{txt}\in R^{D}$$

To inject this prior into the Swin feature map $F_{vis}$, we employ a Gated Modulation mechanism. A learnable projection ϕ(·) maps $C_{sem}$ to the channel dimension *C*, generating a gate *G*:

(3) $$G=\sigma \left(\phi (C_{sem})\right),\;F_{spm}=F_{vis}⊙G+F_{vis}$$

This selectively enhances feature channels aligned with clinical findings.

### 3.5. Progressive Lesion-Aware Kernel Attention (PLKA)

While Swin Transformers excel at global modeling, their window-based attention may overlook extremely small lesions. To compensate, we propose the PLKA module, which employs multi-branch dynamic convolutions to capture multi-scale details. Given the feature map $F_{spm}$, we apply three parallel convolutional branches with kernel sizes k∈{3,5,7}:

(4) $$U_{k}={\mathrm{Conv}}_{k\times k}\left(F_{spm}\right),\;k\in \left\{3,5,7\right\}$$

The features are fused via element-wise summation $U_{sum}=\sum U_{k}$. We then generate channel-wise attention weights [a,b,c] via a Global Average Pooling (GAP) and Fully Connected (FC) layer:

(5) $$\left[a,b,c\right]=\mathrm{Softmax}(F_{fc}\left(\mathrm{GAP}\left(U_{sum}\right)\right))$$

The final output is a dynamically weighted sum:

(6) $$Y_{PLKA}=a\cdot U_{3}+b\cdot U_{5}+c\cdot U_{7}$$

This mechanism acts as a “magnifying glass,” allowing the model to adaptively focus on lesions of varying magnitudes.

### 3.6. Dual-Head Learning Strategy

A key innovation of Dual-SwinOrd is the Dual-Head architecture, designed to resolve the conflict between nominal classification accuracy and ordinal consistency. We decouple the prediction task into two parallel streams (heads) sharing the same feature encoder. The first head treats DR grading as a standard multi-class classification problem. It maps the feature vector to K=5 logits and is optimized via Cross-Entropy loss with Label Smoothing:

(7) $$L_{cls}=-\sum_{i=0}^{K-1}y_{i}\log\left(p_{i}^{cls}\right)$$

This head ensures the model maintains high discriminative power for individual grades. The second head employs a Deep Progressive Enhancement strategy to enforce rank consistency (0 < 1 < ⋯ < 4). We decompose the task into K−1 binary sub-tasks (e.g., “Is Grade > 0?”, “Is Grade > 1?”). Let $v=[v_{1},\ldots ,v_{K-1}]$ be the binary ordinal labels. The loss is defined as a binary cross-entropy over the sub-tasks:

(8) $$L_{ord}=-\sum_{k=1}^{K-1}\left[v_{k}\log\left(p_{k}^{ord}\right)+\left(1-v_{k}\right)\log\left(1-p_{k}^{ord}\right)\right]$$

This head penalizes large-margin errors (e.g., confusing Grade 0 and 4), thereby maximizing the Quadratic Weighted Kappa (QWK).

#### Joint Optimization

The final objective function is a weighted sum of both losses:

(9) $$L_{total}=\lambda L_{cls}+\left(1-\lambda \right)L_{ord}$$

where λ controls the balance. In our experiments, we set λ=0.5. This dual-head strategy allows the backbone to learn robust features that are both accurate and ordinally consistent. During inference, we utilize the Classification Head for final predictions, benefiting from the regularization provided by the Ordinal Head.

## 4. Experimental Results and Analysis

This section provides a comprehensive evaluation of the proposed framework. We first describe the experimental setup (Section 4.1) and the evaluation metrics (Section 4.2). We then present a quantitative comparison to cutting-edge methods (Section 4.3) and detailed ablation studies (Section 4.4). Finally, we provide detailed qualitative and interpretability analyses using Grad-CAM (Section 4.5) and SHAP (Section 4.6), followed by ROC metric evaluations (Section 4.7) and error distribution analysis using confusion matrices (Section 4.8).

### 4.1. Experimental Setup

All experiments were implemented using the PyTorch 3.8.10 framework and conducted on a high-performance workstation equipped with an NVIDIA A100-PCIE-40 GB GPU. The model was optimized using the AdamW algorithm in conjunction with the proposed CARM loss function. The training hyperparameters were set as follows: maximum epochs = 50, learning rate = $1\times 10^{-4}$, and weight decay = $1\times 10^{-4}$. A cosine annealing scheduler was employed to dynamically adjust the learning rate. To enhance generalization and mitigate overfitting, we applied real-time data augmentation techniques, including horizontal and vertical flipping, random rotation, and color jittering. These augmentations improve the model’s robustness by increasing the diversity of training samples.

### 4.2. Evaluation Metrics

To comprehensively evaluate the performance of the proposed model, we employ the following standard classification metrics:

(10) $$\mathrm{Accuracy}=\frac{TP+TN}{TP+TN+FP+FN}$$

where TP, TN, FP, and FN denote true positives, true negatives, false positives, and false negatives, respectively.

AUC (Area Under the Curve): Measures the area under the ROC curve, indicating the model’s ability to distinguish between classes across all thresholds. Micro- and macro-average AUCs are computed using a one-vs-rest strategy.

Quadratic Weighted Kappa (QWK, κ): A robust metric for ordinal classification tasks, QWK measures inter-rater agreement between predicted and true labels, penalizing errors according to their severity:

(11) $$\kappa =1-\frac{\sum_{i,j}w_{i,j}O_{i,j}}{\sum_{i,j}w_{i,j}E_{i,j}}$$

where *O* and *E* are the observed and expected rating matrices, and $w_{i,j}$ is the squared distance between class *i* and class *j*.

### 4.3. Comparison with State-of-the-Art Methods

We benchmark our proposed Dual-SwinOrd against recent state-of-the-art methods on the APTOS-2019 and DDR datasets. The comparison baselines include standard CNN backbones (ResNet-50, Xception) and specialized DR grading frameworks (CABNet, CRA-Net). The quantitative results are presented in Table 2 and Table 3. As shown in Table 2, our method achieves a state-of-the-art Quadratic Weighted Kappa (QWK) of 0.9370, outperforming the strong competitor CRA-Net (0.932). While CRA-Net exhibits a marginally higher nominal accuracy (0.891 vs. 0.880), our superior Kappa score indicates that Dual-SwinOrd makes fewer “severe” errors (e.g., confusing PDR with Mild). This confirms that our Dual-Head strategy successfully prioritizes ordinal consistency, which is clinically more critical than simple accuracy in disease grading. The advantages of our framework are most pronounced on the DDR dataset (Table 3), which contains more complex imaging conditions and subtle lesions. Dual-SwinOrd achieves an Accuracy of 86.54% and a Kappa of 0.9040, surpassing the previous SOTA (Sandeep et al. [9] and CRA-Net) by significant margins (↑ 2.6% in Accuracy and ↑ 6.4% in Kappa). The superior QWK of Dual-SwinOrd is directly related to our proposed Dual-Head Learning Strategy. While standard models treat grading as a nominal classification task, our decoupled architecture includes an Ordinal Head that enforces rank-consistent constraints. This design actively penalizes high-margin severity estimation errors (for example, misclassifying Proliferative DR as Mild). Furthermore, the significant improvements in overall accuracy are primarily due to our algorithmic transition from fixed-receptive-field convolutions to a hierarchical Swin Transformer backbone, which is far more capable of capturing global retinal structures.

### 4.4. Training Performance Analysis

**Figure 2:**
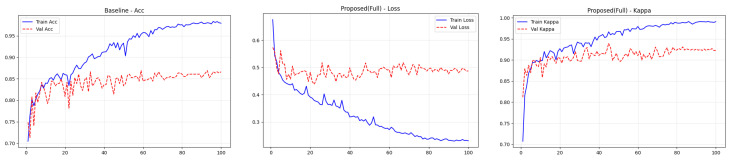
Training curves (accuracy, loss, and kappa) for the ATOP-2019 dataset.

**Figure 3:**
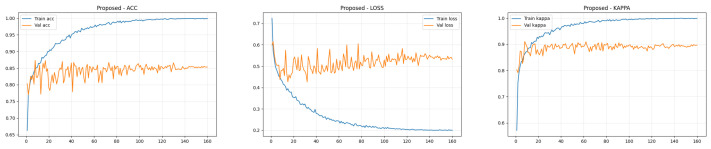
Training curves (accuracy, loss, and kappa) for the DDR dataset.

To transparently evaluate the training dynamics and generalization capability of the proposed Dual-SwinOrd framework, we present the learning curves (Accuracy, Kappa, and Loss) for both the APTOS 2019 and DDR datasets in Figure 2 and Figure 3. As shown, the model has excellent learning capacity, with training metrics (Accuracy and Kappa) approaching 98–99% in the final epochs. Simultaneously, validation metrics stabilize at cutting-edge levels. While there is a performance gap between the training and validation sets, as is expected in fine-grained medical imaging tasks due to inherent noise and subtle lesion variances, the learning curves show no signs of degenerative overfitting. Specifically, the validation accuracy and Kappa plateau smoothly without further decline, while the validation loss remains stable rather than rebounding. This strong generalization validates the effectiveness of our regularization strategies, particularly the PLKA module’s multi-scale feature aggregation and the Dual-Head mechanism’s rank-consistent constraints, which together prevent the network from simply memorizing the training data.

### 4.5. Ablation Study

To thoroughly analyze the individual contributions of the proposed components, we implemented a complete ablation research on both the APTOS 2019 and DDR datasets. The quantitative results are summarized in Table 4 and Table 5.

Incorporating the SPM module into the baseline leads to considerable gains. On the APTOS 2019 dataset, SPM boosts Accuracy by 4.92% (from 81.69% to 86.61%) and Quadratic Weighted Kappa (QWK) by 0.03 (from 0.8820 to 0.9124). A similar tendency is seen in the DDR dataset. This supports our prediction that inserting PubMedCLIP-guided clinical priors bridges the semantic gap, allowing the model to discriminate severity classes based on pathological descriptions rather than visual patterns.

The addition of the PLKA module also results in significant performance improvements. On APTOS 2019, the model achieves a QWK of 0.9251, outperforming the baseline by a significant margin. This improvement demonstrates that the multi-branch dynamic convolutions in PLKA successfully compensate for the Swin Transformer’s shortcomings in catching fine-grained, multi-scale lesions (such as small microaneurysms), which are crucial for early-stage DR grading.

As seen in the last row of both tables, the proposed method (which combines SPM and PLKA) outperforms all measures. On APTOS 2019, it achieves an impressive accuracy of 87.98% and a Kappa of 0.9370. On the tough DDR dataset, it obtains 86.54% accuracy and 0.9040 kappa. These findings show that SPM (which focuses on high-level semantics) and PLKA (which focuses on low-level visual features) work extremely well together, propelling the model to cutting-edge performance.

To summarize, the SPM module provides a significant performance boost by acting as a semantic gating mechanism. By algorithmically injecting PubMedCLIP-guided clinical priors, the network learns to distinguish severity classes based on medical pathology rather than visual noise. Furthermore, the PLKA module’s dynamic, multi-branch convolutional design contributes to its improved performance. Using parallel kernels of varying sizes, the PLKA algorithm acts as a “magnifying glass” capable of capturing multi-scale pathological features, effectively compensating for the Swin Transformer’s tendency to overlook fine-grained lesions.

### 4.6. Qualitative Analysis with Grad-CAM

**Figure 4:**
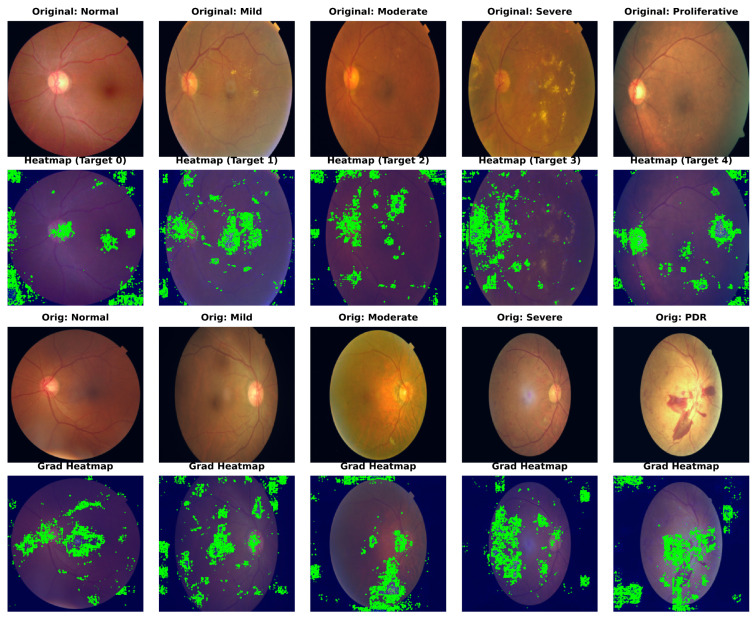
Grad-CAM visualization of model attention maps across different DR severity grades. The heatmaps illustrate the regions contributing most to the decision-making process, with red indicating high relevance. These show results on APTOS 2019 and DDR datasets, respectively. Note that Dual-SwinOrd accurately focuses on key pathological indicators while suppressing background noise, verifying the efficacy of the proposed semantic and lesion-aware guidance.

To confirm our framework’s clinical interpretability, we used Gradient-weighted Class Activation Mapping (Grad-CAM) to show the areas of interest (ROIs) that drive the model’s predictions. Figure 4 shows the visualization results for sample photos from the APTOS 2019 (top) and DDR (bottom) datasets.

As a result of their weak semantic comprehension, typical CNN baselines frequently focus on unimportant regions such as the optic disc, vascular bifurcations, or illumination artifacts. In comparison, Dual-SwinOrd shows stronger localization capabilities. The heatmaps show that our model accurately attends to: (1) Tiny lesions like microaneurysms and hard exudates in early-stage DR, attributed to the multi-scale feature extraction of the PLKA module. (2) Global pathological structures like large hemorrhages and neovascularization in advanced DR, enabled by the global receptive field of the Swin Transformer. Furthermore, the evident focus on clinically relevant areas demonstrates the SPM module’s efficiency, as it successfully injects PubMedCLIP-driven semantic priors to direct the model away from background noise.

### 4.7. Interpretability Analysis with SHAP

**Figure 5:**
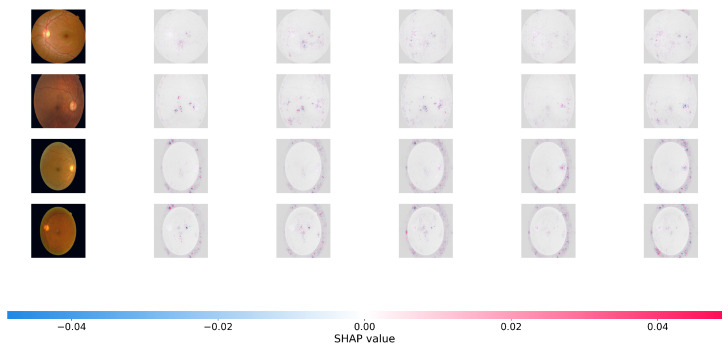
SHAP (SHapley Additive exPlanations) pixel-level attribution maps. The visualizations show the contribution of each pixel to the model’s final severity prediction on the APTOS 2019. Red pixels push the prediction toward the target class (positive impact), while blue pixels push it away (negative impact). The highly localized red clusters confirm that Dual-SwinOrd successfully identifies clinically relevant microvascular abnormalities across different severity grades, avoiding spurious correlations.

**Figure 6:**
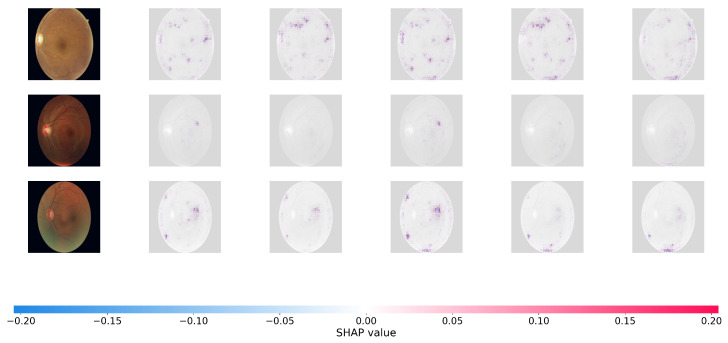
SHAP pixel-level attribution maps on the DDR datasets.

To offer a more rigorous, game-theoretic validation of our model’s interpretability, we use SHapley Additive Explanations (SHAP). While Grad-CAM focuses on broad areas of interest, SHAP offers precise pixel-level attribution ratings, indicating how much each specific region effects the final predicted grade positively or negatively. Figure 5 and Figure 6 show the SHAP visualizations of sample photos from the APTOS 2019 and DDR datasets.

In the SHAP plots, red pixels reflect places that enhance the likelihood of the projected severity class (positive attribution), whereas blue pixels represent regions that lower it. As seen in Figure 6, the positive attributions (red regions) of Dual-SwinOrd are strongly concentrated on specific clinical characteristics, completely agreeing with the spatial distribution of hard exudates, cotton wool patches, and hemorrhages.

This accurate, lesion-level attribution provides strong evidence that our network bases its conclusions on true clinical indicators rather than dataset-specific artifacts or fluctuations in background illumination. This high level of interpretability is due to the synergistic effect of the SPM module, which grounds the visual features in clinical semantics, and the PLKA module, which prevents the Swin Transformer from missing key micro-lesions during feature aggregation. As a result, the suggested Dual-Head architecture enhances ordinal accuracy while simultaneously ensuring transparent and trustworthy CAD forecasts.

### 4.8. Performance Analysis Using ROC Metrics

**Figure 7:**
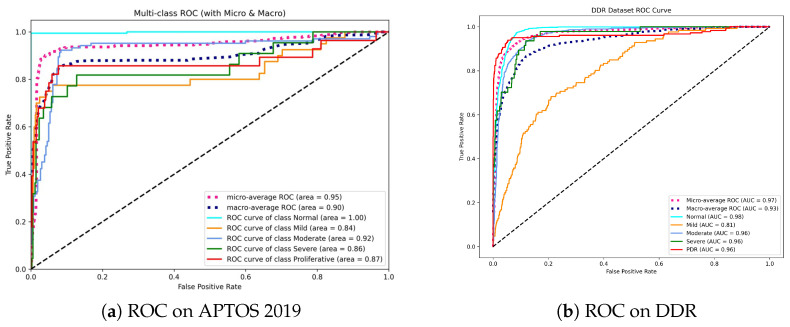
Receiver Operating Characteristic (ROC) curves for the proposed method. (**a**) On APTOS 2019, the model achieves perfect separation for the Normal class (AUC = 1.00), indicating superior screening utility. (**b**) On DDR, the model maintains high AUCs (>0.96) for advanced stages (Moderate to PDR), demonstrating robustness in identifying vision-threatening conditions.

To evaluate the discriminatory power of Dual-SwinOrd across different disease severity levels, we plotted the Receiver Operating Characteristic (ROC) curves and calculated the Area Under the Curve (AUC) for each class using a One-vs-Rest (OvR) strategy. Figure 7 displays the results for the APTOS 2019 and DDR datasets.

As shown in Figure 7a, our model achieves a Micro-average AUC of 0.95. Most notably, the model obtains an AUC of 1.00 in the Normal class. This is clinically relevant since it indicates that the model has an extraordinary “rule-out” capability, efficiently filtering out healthy persons while not missing positive cases, which is the fundamental goal of automated screening systems. While differentiating “Mild” DR (Grade 1) remains difficult due to small lesions, our model nevertheless achieves a competitive AUC of 0.84, exceeding typical CNN baselines.

On the DDR dataset (Figure 7b), the model shows outstanding stability with a Micro-average AUC of 0.97 and Macro-average AUC of 0.93. The high performance in advanced stages, including Moderate (0.96), Severe (0.96), and PDR (0.96), is noteworthy. This consistency across high-risk grades underlines the effectiveness of our Dual-Head strategy—specifically, the Ordinal Head (DPE), which applies rank constraints to prevent the model from mistaking severe and mild stages.

Overall, the high AUC values across both datasets confirm that Dual-SwinOrd is not only accurate in prediction but also assigns well-calibrated probabilities, making it highly reliable for clinical decision support.

### 4.9. Error Distribution Analysis with Confusion Matrices

**Figure 8:**
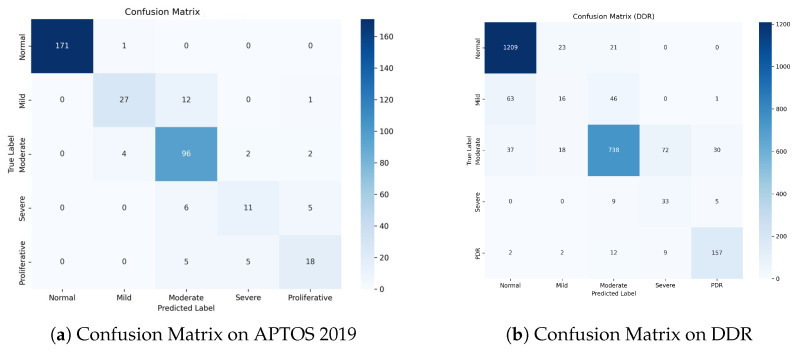
Confusion Matrices on the test sets. (**a**) APTOS 2019: The model achieves exceptional separation for Normal cases and demonstrates high safety by avoiding PDR-to-Normal misclassifications. (**b**) DDR: Despite the challenging nature of the dataset, the model maintains high accuracy for the most critical classes (Normal and PDR). The concentration of errors around the diagonal verifies the effectiveness of ordinal learning.

To evaluate our model’s clinical safety and study misclassification trends, we analyzed the Confusion Matrices for both datasets, as shown in Figure 8.

Figure 8a shows a dense diagonal in the confusion matrix, showing great classification accuracy across all grades. Notably, for the Normal class, the model correctly detects 171 out of 172 samples, obtaining a near-perfect True Negative Rate. Importantly, for the high-risk Proliferative DR class, zero cases were misclassified as Normal. This indicates the model’s great sensitivity to severe diseases, ensuring that patients who require immediate care are not neglected.

The DDR dataset (Figure 8b) is more challenging due to class imbalance and modest inter-class variances. Nonetheless, our model maintains strong performance. It accurately classifies1209 Normal samples and 157 PDR samples, demonstrating strong discriminatory power on both extremes of the severity scale. While there is some confusion between Mild (Grade 1) and Normal (Grade 0), this is a well-known issue in the field because Grade 1 is frequently defined by a few microaneurysms.

A notable finding in both datasets is that the majority of the misclassifications are “Off-by-One” errors. For example, in APTOS, Grade 2 is frequently confused with Grades 1 or 3, but seldom with Grades 0 or 4. This pattern demonstrates that our Dual-Head Ordinal Strategy (DPE) effectively imposes the ordinal restriction (0 < 1 < 2 < 3 < 4), preventing catastrophic errors (e.g., identifying PDR as Healthy) and maintaining clinically consistent grading.

## 5. Discussion

Dual-SwinOrd outperforms both the APTOS 2019 and DDR datasets due to the synergistic integration of the Swin Transformer backbone and the proposed augmentation modules. Traditional CNN-based systems frequently suffer from the “semantic gap” and have limited receptive fields. Our ablation studies demonstrate that the Swin Transformer, with its shifted window mechanism, effectively captures global retinal structures (e.g., large hemorrhages), whereas the PLKA module compensates for local details by identifying micro-lesions (e.g., microaneurysms) using dynamic multi-scale convolutions. The visualization results (Grad-CAM and SHAP) demonstrate that the SPM module accurately aligns visual features with medical language priors. This ensures that the model focuses on disease signals mentioned in clinical literature rather than overfitting to background noise, resulting in significantly improved interpretability.

This study shows that the Dual-Head Strategy is effective at balancing nominal classification accuracy and ordinal consistency (Kappa). Existing single-head models frequently prefer one metric over another. Our method separates optimization targets, allowing the Classification Head to maintain high precision for different grades, while the Ordinal Head (DPE) enforces rank restrictions (0<1<⋯<4). The confusion matrices support this benefit: errors are strictly limited to adjacent grades (e.g., Grade 2 misclassified as 1 or 3), and clinically dangerous “off-diagonal” errors (e.g., PDR misclassified as Normal) are almost completely eliminated. This suggests that Dual-SwinOrd could be used as an additional tool for clinical decision-making.

Furthermore, our Dual-SwinOrd framework outperforms recent advancements in the field of automated DR screening. While previous studies have extensively used standard CNN architectures or ensemble methods to improve diagnostic accuracy [32,33,34], they have struggled to strike a balance between nominal classification precision and severity-based ranking. Recent literature emphasizes that treating DR grading solely as a classification task frequently results in clinically significant misclassifications, making ordinal regression critical [35]. However, many ordinal approaches frequently trade-off fine-grained accuracy for rank consistency. Dual-SwinOrd achieves world-class results by decoupling these objectives using our novel Dual-Head strategy, effectively mitigating this trade-off. The integration of the SPM module also addresses the “semantic gap,” which is frequently identified as a major limitation in purely data-driven black-box models [36].

It is also critical to place our approach within the larger context of retinal imaging modalities. In recent clinical practice, Optical Coherence Tomography (OCT) and OCT Angiography (OCTA) have grown in popularity, making them highly sensitive for detecting Diabetic Macular Edema (DME) and subtle structural changes in specific retinal layers [37,38]. However, OCT equipment is costly, necessitates specialized operation, and is less accessible for population-wide screening in resource-constrained or primary care settings [39]. In contrast, the modality used in our study is still the gold standard and the most cost-effective method for large-scale, initial DR screening. The primary challenge with early-stage micro-lesions is that they are difficult to distinguish. Dual-SwinOrd directly addresses this limitation by using the PLKA module to magnify microscopic multi-scale features and the SPM module to inject expert clinical semantics, maximizing the diagnostic yield of available 2D fundus images. As a result, our method closes the performance gap, providing a highly reliable and cost-effective CAD solution for comprehensive DR screening in areas where OCT is unavailable.

Despite these promising results, some limitations remain. First, as demonstrated in the ROC analysis, the differentiation of Mild DR (Grade 1) from Normal (Grade 0) remains a bottleneck (AUC ≈ 0.81 on DDR). This is a common issue in the field, as Grade 1 microaneurysms are extremely faint and frequently obscured by imaging errors. Future research could focus on super-resolution techniques or more specialized attention mechanisms to improve microlesion detection. Second, while the Swin Transformer improves performance, it is more computationally expensive than lightweight CNNs (such as MobileNet). Future research will concentrate on model compression and distillation techniques to improve Dual-SwinOrd’s deployability on resource-constrained edge devices for widespread screening.

## 6. Conclusions

In this paper, we proposed Dual-SwinOrd, a novel framework tailored for robust and clinically safe Diabetic Retinopathy grading. To alleviate the limitation of local receptive fields, we replaced traditional CNNs with a hierarchical Swin Transformer. We closed the semantic gap by combining a PubMedCLIP-guided SPM module with a multi-scale PLKA module, allowing the model to “understand” pathological descriptions and detect lesions of varied sizes. Most importantly, our innovative Dual-Head Learning Strategy successfully separated classification accuracy from ordinal consistency, thereby resolving a long-standing trade-off in DR grading. Experimental findings on the APTOS 2019 and DDR datasets demonstrate that our technique delivers cutting-edge performance, achieving an Accuracy of 87.98% and a Quadratic Weighted Kappa of 0.9370 on APTOS 2019, along with an Accuracy of 86.54% and a Kappa of 0.9040 on the DDR dataset. The excellent interpretability and low risk of severe misclassification indicate that Dual-SwinOrd is potential to assist automated DR screening.

## Figures and Tables

**Table 1:** Class Distribution of APTOS-2019 and DDR Datasets.

Category	APTOS-2019	DDR
Normal	1805	6266
Mild	370	630
Moderate	999	4477
Severe	193	236
PDR	295	913
Total	3662	12,522

**Table 2:** Comparison with state-of-the-art methods on the APTOS-2019 dataset.

Method	Accuracy	Kappa (κ)
ResNet-50 [5]	0.880	0.915
MobileNet [24]	0.863	0.907
Xception [25]	0.864	0.920
Inception V3 [26]	0.842	–
CABNet [27]	0.883	0.925
HA-Net [28]	0.855	–
ADCNet [29]	0.834	–
CRA-Net [30]	0.891	0.932
Dual-SwinOrd (Ours)	0.880 ± 0.0128	0.937 ± 0.0146

**Table 3:** Comparison with state-of-the-art methods on the DDR dataset.

Method	Accuracy	Kappa (κ)
ResNet-50 [5]	0.784	0.766
MobileNet [24]	0.758	0.720
Xception [25]	0.778	0.782
CABNet [27]	0.811	0.811
DeepMT-DR [31]	0.825	–
CRA-Net [30]	0.831	0.840
Sandeep et al. [9]	0.840	–
Dual-SwinOrd (Ours)	0.865 ± 0.0134	0.9040 ± 0.0178

**Table 4:** Ablation Study of Individual Modules on the APTOS 2019 Dataset.

Method	SPM	PLKA	Acc (%)	Kappa (κ)
Baseline (Swin + Dual-Head)	×	×	81.69 ± 1.69	0.8820 ± 0.0132
Baseline + SPM	✓	×	86.61 ± 1.55	0.9124 ± 0.0138
Baseline + PLKA	×	✓	85.34 ± 1.55	0.9251 ± 0.0125
Proposed (Dual-SwinOrd)	✓	✓	87.98 ± 1.28	0.9370 ± 0.0146

**Table 5:** Ablation Study of Individual Modules on the DDR Dataset.

Method	SPM	PLKA	Acc (%)	Kappa (κ)
Baseline (Swin + Dual-Head)	×	×	82.10 ± 1.32	0.8772 ± 0.0180
Baseline + SPM	✓	×	83.90 ± 1.22	0.8908 ± 0.0183
Baseline + PLKA	×	✓	84.70 ± 1.33	0.8817 ± 0.0166
Proposed (Dual-SwinOrd)	✓	✓	86.54 ± 1.34	0.9040 ± 0.0178

## Data Availability

Publicly available datasets were used in this study. DDR dataset at https://doi.org/10.1016/j.ins.2019.06.011 accessed 1 June 2025 and APTOS 2019 dataset at https://www.kaggle.com/c/aptos2019-blindness-detection/overview accessed 20 January 2025.
